# Properties of African Cassava Mosaic Virus Capsid Protein Expressed in Fission Yeast

**DOI:** 10.3390/v8070190

**Published:** 2016-07-08

**Authors:** Katharina Hipp, Benjamin Schäfer, Gabi Kepp, Holger Jeske

**Affiliations:** Department of Molecular Biology and Plant Virology, Institute of Biomaterials and Biomolecular Systems, University of Stuttgart, Pfaffenwaldring 57, D-70550 Stuttgart, Germany; benjamin.schaefer@bio.uni-stuttgart.de (B.S.); gabi.kepp@bio.uni-stuttgart.de (G.K.); holger.jeske@bio.uni-stuttgart.de (H.J.)

**Keywords:** geminivirus, capsid protein, CP, fission yeast, ectopic expression, DNA binding assay

## Abstract

The capsid proteins (CPs) of geminiviruses combine multiple functions for packaging the single-stranded viral genome, insect transmission and shuttling between the nucleus and the cytoplasm. African cassava mosaic virus (ACMV) CP was expressed in fission yeast, and purified by SDS gel electrophoresis. After tryptic digestion of this protein, mass spectrometry covered 85% of the amino acid sequence and detected three N-terminal phosphorylation sites (threonine 12, serines 25 and 62). Differential centrifugation of cell extracts separated the CP into two fractions, the supernatant and pellet. Upon isopycnic centrifugation of the supernatant, most of the CP accumulated at densities typical for free proteins, whereas the CP in the pellet fraction showed a partial binding to nucleic acids. Size-exclusion chromatography of the supernatant CP indicated high order complexes. In DNA binding assays, supernatant CP accelerated the migration of ssDNA in agarose gels, which is a first hint for particle formation. Correspondingly, CP shifted ssDNA to the expected densities of virus particles upon isopycnic centrifugation. Nevertheless, electron microscopy did not reveal any twin particles, which are characteristic for geminiviruses.

## 1. Introduction

African cassava mosaic virus (ACMV) is a small plant pathogenic virus belonging to the *Begomovirus* genus of the *Geminiviridae* family [1]. It is one of the most prevalent pathogens of cassava in Africa [2,3,4]. Its capsid protein (CP) plays a key role in whitefly-transmission [5,6,7,8,9,10,11,12], determines the exceptional twin shape of the virions [13,14], and is expressed from the genes (AV1, V1) of the viral strand of DNA A for bipartite or DNA A-like components for monopartite geminiviruses, respectively. The three-dimensional structure of ACMV virions resolved by electron cryomicroscopy revealed pentameric capsomeres in two incomplete T = 1 icosahedra [13]. Geminiviral CPs adopt the eight-stranded β-barrel fold found in many small icosahedral viruses ([13,14,15] and references therein). Detailed studies on the assembly process of these virions are still lacking. CP pentamers were proposed to be substructures during assembly [16] and were detected during in vitro disassembly of ACMV particles [17]. The tomato yellow leaf curl virus (TYLCV) CP showed self-interaction in yeast, two-hybrid assays and in tobacco protoplasts, and the N-terminus of one CP molecule was found to interact with the C-terminus of a second one [18,19]. Additionally, mung bean yellow mosaic India virus (MYMIV) CP has been reported to interact with the viral replication-initiator protein (Rep) [20] which might indicate a coupling of assembly and replication.

Several geminiviral CPs have been examined by in vitro binding studies after bacterial expression. CPs can bind ssDNA as well as dsDNA in a cooperative and sequence non-specific manner [21,22,23,24,25]. Amino acid exchanges localized a DNA binding domain to the N-terminus [23,25,26,27]. Three regions of ACMV CP—at the N-terminus, within the central part, and the C-terminus—contain nuclear localization signals (NLS) [28]. The central region is also important for nuclear export [28]. Deleting the NLS regions inhibited the formation of virus particles in vivo [29]. Thus, geminiviral CPs are multifunctional proteins [28,29,30,31,32,33].

The bacterially expressed CPs formed inclusion bodies and had to be dissolved under highly denaturing urea concentrations [20,21,23,24,25,34]. In order to overcome the corresponding folding problems, and considering that the authentic compartment for CP assembly is the nucleus, ACMV CP was expressed in fission yeast under the control of an inducible promoter to study its’ in vitro assembly with ssDNA.

## 2. Materials and Methods

### 2.1. Construction of Expression Plasmid

The ACMV AV1 open reading frame (ORF) was amplified by PCR (3 min, 94 °C; 25 cycles 30 s 94 °C, 30 s 52 °C, 1 min 72 °C; 10 min 72 °C) using Taq-DNA-polymerase (Qiagen, Hilden, Germany), pUC19:APA-9 (Dr. Rob W. Briddon, Faisalabad, Pakistan, containing the sequence of ACMV-[Nigeria-Ogo]; AJ427910) as template, forward 5′-ACCCGGGTCGACATGTCGAAGCGACCAGGA-3′ and reverse primer 5′-ACCCGGGTTAATTGCCAATACTGTCATA-3′ adding *Sma*I and *Sal*I (underlined) restriction sites. Fragments were inserted into pGEM-T (Promega Corporation, Mannheim, Germany), transformed into *E. coli*, excised with *Sma*I and ligated into *Sma*I-digested pESP1-pREP2 [35] yielding pESP1-pREP2:AV1. The correctness of the construct was confirmed using Li-Cor DNA-Sequencer Modell 4000L (Li-Cor Bioscience GmbH, Bad Homburg, Germany). Compared to the database entry, the pUC19-APA9 clone showed four nucleotide substitutions (C164A, T165G resulting in a T55M amino acid exchange, and silent A390G, G399T; numbering according to AV1 sequence).

### 2.2. Expression in Schizosaccharomyces pombe (S. pombe)

Either pESP1-pREP2:AV1 or pESP1-pREP2 as a vector control were transformed into *S. pombe* SP-Q01 as described [36]. Small-scale induction of protein expression was carried out as detailed previously [36], but using Edinburgh minimal medium (EMM) to induce protein expression, or EMM supplemented with 5 µM thiamine, to repress it. For Western blots, 1–1.5 mL of a cell culture were harvested at 3, 6, 9, 12, 15, and 18 h post induction (hpi) by centrifugation (1000× *g*, 5 min). The cell pellets were frozen in liquid nitrogen and stored at −80 °C until use. For density gradient centrifugation, size-exclusion chromatography, solubilization experiments and binding assays, 10 mL of the cultures were harvested 9 hpi, washed once with 10 mL of cold PBS (140 mM NaCl, 2.7 mM KCl, 10 mM Na_2_HPO_4_, 1.8 mM KH_2_PO_4_, pH 7.2), centrifuged (1000× *g*, 5 min), and stored at −80 °C.

### 2.3. Preparation of Cell Extracts

The cell extracts were prepared as described [36] with slight modifications: cells from 1–1.5 mL or 10 mL cultures were resuspended in 50–150 µL or 500 µL PBST-PI and disrupted by either vortexing (4 °C, 5–7 min in 15 s intervals and intermitting cooling on ice) or in a Fast Prep 24 device (MP Biomedicals, Illkirch, France; 10 cycles at 6 m·s^−1^ and 15 s with intermitting cooling on ice).

### 2.4. Western Blot Analysis

The protein samples were separated in 12.5% SDS-polyacrylamide gels (SDS-PAGE) according to Laemmli [37] Samples were denatured in loading buffer for 5 min at 95 °C, and iodacetamide was subsequently added to a final concentration of 140 mM. The gels were stained with 0.5% Coomassie Brilliant Blue R 250 Powder (SERVA Electrophoresis GmbH, Heidelberg, Germany) in 50% EtOH, 7% acetic acid, or they were semi-dry blotted [38] onto nitrocellulose using a Protran Nitrocellulose Transfer Membrane (Whatman Schleicher & Schuell, Dassel, Germany). CP was detected using 1:1000 diluted polyclonal rabbit antiserum raised against partially purified ACMV particles kindly (provided by Dr. J. Stanley, Norwich, UK, [39]) or AS-0421 (Dr. S. Winter, DSMZ, Braunschweig, Germany), alkaline phosphatase conjugated goat anti-rabbit antibodies (Rockland Immunochemicals, Inc., Gilbertsville, PA, USA), and nitro-blue tetrazolium choride, 5-bromo-4-choro-3′-indolylphosphate (NBT/BCIP).

### 2.5. Mass Spectrometry

The extract from induced yeast cells was separated by SDS-PAGE to excise the CP band after Coomassie-staining. In gel trypsin-digestion and NanoLC-ESI-MS/MS was preformed by the Core Facility of the Life Science Center, University of Hohenheim, Stuttgart, Germany.

### 2.6. Density Gradient Centrifugation

Cs_2_SO_4_ (35% *w*/*v* final concentration) was added to supernatant or pellet fractions of the cell extracts from 10 mL cultures. After a pre-centrifugation (20 min, 16,340× *g*, 4 °C; Sorvall RC 5C, Hb6 rotor) for the pellet fraction, in order to remove insoluble material, both supernatants were used for the isopycnic gradient centrifugation (17 h, 50,000 rpm, 4 °C, Beckman L7-65, VTi65.1 rotor). Fractions from the bottom of the tube in 350 to 400 µL-aliquots were analyzed by UV-absorption, enzyme-linked immunosorbent assay (ELISA), Western blotting, and refractometry (Zeiss, Oberkochen, Germany). For SDS-PAGE, 100 µL aliquots were precipitated by adding 20 µL 1 mg·mL^−1^ yeast RNA (Roche Applied Sciences, Mannheim, Germany), 1/10 vol. 3 M Na-acetate pH 4.8 and 2 vol. ethanol, and washed twice with 70% ethanol and resuspended in 20 µL water, a quarter of which was applied per lane.

### 2.7. Solubilization of Capsid Proteins (CPs)

Cell extract pellets were incubated with increasing concentrations of NaCl (0.2, 0.3, 0.4, 0.5, 0.6, 0.7, 0.8, 0.9, 1.0, 1.1, 1.2, 1.5 M f. c.) for 15 min at 4 °C. Aliquots were taken and centrifuged (5 min, 12,000× *g*, 4 °C). The pellets were resuspended in the same volume as for the respective supernatants and both fractions were analyzed by ELISA. For pH-dependent solubilization, cell extract pellets were incubated with increasing volumes of NaH_2_PO_4_ (pH 7.0–4.4) or Na_2_HPO_4_ (pH 7.0–8.1), and processed similarly.

### 2.8. Size-Exclusion Chromatography

Superose 6 HR 10/30 was equilibrated with 50 mM sodium phosphate pH 7.0 plus 0.6 M NaCl using an FPLC system controlled by the FPLC director software (version 1.03) or an ÄKTA basic (GE Healthcare, Munich, Germany). The supernatant samples (0.5–3.5 mg total protein) were centrifuged (10 min, 10,000× *g*) before application and collection of 300 µL fractions. For DNA removal, the samples were incubated with DNaseI and 2 mM MgCl_2_ (Roche Applied Sciences, Mannheim, Germany; 30 min, 37 °C) prior to chromatography. To analyze the influence of reducing agents, 1 mM DTT was added to the samples and analyzed on the column equilibrated with a buffer as before, but containing 1 mM DTT. For SDS-PAGE and Western blotting, aliquots of the fractions were prepared as described in [29].

### 2.9. Enzyme-Linked Immunosorbent Assay (ELISA)

CP-containing fractions from density gradient centrifugation or chromatography were identified by ELISA: aliquots (50 µL) in 100 µL PBS containing 0.05% Tween-20 and 2% polyvinylpyrrolidone (PVP 40,000, Sigma, Taufkirchen, Germany) or in 100 µL coating buffer (15 mM Na_2_CO_3_, 35 mM NaHCO_3_ pH 9.6) were incubated overnight at 4 °C or 3 h at 37 °C in a 96-well microtiter, high binding plate (Greiner Bio-One International AG, Frickenhausen, Germany). After one washing step with PBS supplemented with 0.05% Tween-20, the wells were blocked with 150 µL of 3% gelatin in PBS three times for 10 min, washed again and incubated with 100 µL 1:5000 diluted polyclonal antiserum raised against purified ACMV particles or AS-0421 for 2–3 h at 37 °C. A secondary antibody (100 µL; alkaline phosphatase conjugated goat anti-rabbit, 1:5000 dilution) was added for (1 h, 37 °C). After washing three times for 3 min, 100 µL 1 mg·mL^−1^ *p*-nitrophenyl phosphate (PNPP, SERVA Electrophoresis GmbH, Heidelberg, Germany) in 9.7% (*v*/*v*) diethanolamine pH 9.8 were added and the reaction was monitored as at A_405_ (subtracted by A_620_; TECAN Spectrafluor Plus, TECAN, Crailsheim, Germany).

### 2.10. Preparation of ssDNA

Constructs to yield the viral (v) or the complementary (c) strand of ACMV-N DNA A as ssDNA were prepared by releasing fragments with *Hin*dIII/*Kpn*I or *Hin*dIII/*Xba*I from plasmid pUC19-APA9 and inserting them into *Hin*dIII/*Kpn*I or *Hin*dIII/*Xba*I cut pBluescript II SK(-) to obtain phagemids pBlue:ACMVA(v) and pBlue:ACMVA(c). To design an additional circular ssDNA comprising the ACMV-N common region (CR) with approximately the size of the viral genomic components (pBlue:CR250), pBluescript SK(-) was cut with *Sap*I and *Bss*HII, in order to remove the multiple cloning site and flanking regions, and then treated with Klenow Fragment (New England BioLabs GmbH, Frankfurt, Germany; 15 min, 25 °C). The CR fragment obtained by PCR (5 min, 95 °C; 25 cycles 30 s 95 °C, 40 s 57 °C, 3 min 72 °C; 10 min 72 °C) using Pfu-DNA-polymerase (Fermentas GmbH, St. Leon-Rot, Germany), pUC19:APA-9 as the template, forward primer 5′-CTCAACTAGAGACACTCTTGAGC-3′ and reverse primer 5′-GTGGCCCACCACTAATA CATAAC-3′ was ligated to the modified vector (T4 DNA Ligase; New England BioLabs GmbH, Frankfurt, Germany). These new constructs, as well as pCLV1.3A, a bitmer of DNA A of ACMV-Kenya [40,41], were transformed into *E. coli* XL1-Blue MRF’ (Stratagene GmbH, Heidelberg, Germany) and bacteria were cultivated at 37 °C and 180 rpm to an OD_600_ of 0.3 before they were infected with a helper phage (R408; Stratagene GmbH, Heidelberg, Germany) with a multiplicity of infection of 10 and grown further (30 min, 180 rpm, 37 °C; followed by 6 h, 270 rpm). Cells were removed by two centrifugations (15 min, 12,000× *g*, room temperature). Phages in the supernatants were precipitated by polyethylene glycol (PEG, 1/4 vol. 20% PEG 6000 in 3.5 M ammonium acetate overnight, 4 °C) followed by centrifugation (15 min, 12,000× *g*, room temperature), resuspended in 10 mM TrisHCl pH 8.0 containing 1 mM EDTA, and stored at −20 °C. For binding assays, phage ssDNA was prepared by phenol/chloroform extraction, precipitation with ethanol and resuspension in water.

### 2.11. Binding Assays

#### 2.11.1. Experimental Set 1

Yeast extracts (5–25 µL; 3 mg total protein from CP-expressing or vector control cells) were incubated with ssDNA (100–150 ng; 1–2 h, 4 °C under gentle agitation). Complexes were precipitated by adding PEG 6000 and NaCl (f. c. 4%; 0.2 M, respectively) overnight at 4 °C. After centrifugation (20 min, 16,340× *g*, 4 °C), the pellets were resuspended in 18 µL 0.1 M sodium borate pH 8.0 plus 2 mM EDTA, to which 2 µL loading buffer (25% Ficoll, 0.25% bromophenol blue, 0.025 M EDTA) were added for separation on 0.7% agarose gels in 1× TBE (89 mM Tris-HCl, 89 mM boric acid, 2 mM EDTA, 2 h, 4 V·cm^−1^). The gels were stained with 0.5 µg·mL^−1^ ethidium bromide, followed by blotting onto nylon (Hybond N+, GE Healthcare, Munich, Germany) or nitrocellulose using Protran Nitrocellulose Transfer Membranes (Whatman, Schleicher & Schuell, Dassel, Germany) to detect DNA or protein, respectively. Alkaline transfer [42] for Southern blotting or transfer using a buffer [38] supplemented with 0.1% SDS for Western blotting were used. DNA was hybridized with a digoxigenin-labeled ACMV DNA A probe (DIG High Prime, Roche Applied Sciences, Mannheim, Germany), and visualized using alkaline phosphatase conjugated anti-DIG antibodies and chloro-5-substituted adamantyl-1,2-dioxetane phosphate (CSPD, Roche Applied Sciences, Mannheim, Germany). CP on nitrocellulose membranes was detected by incubation with AS-0421 (1:1000), and NBT/BCIP as described above.

#### 2.11.2. Experimental Set 2

The pellet fractions from CP-expressing or vector control extracts were incubated with ssDNA (200 ng; 1–2 h, 4 °C; under gentle agitation) and separated by density gradient centrifugation as described above. CP containing fractions were identified by ELISA and DNA by dot blot hybridization using nylon membranes and a DIG-labeled pBluescript probe (DIG High Prime, Roche Applied Sciences, Mannheim, Germany).

## 3. Results

**Figure 1:**
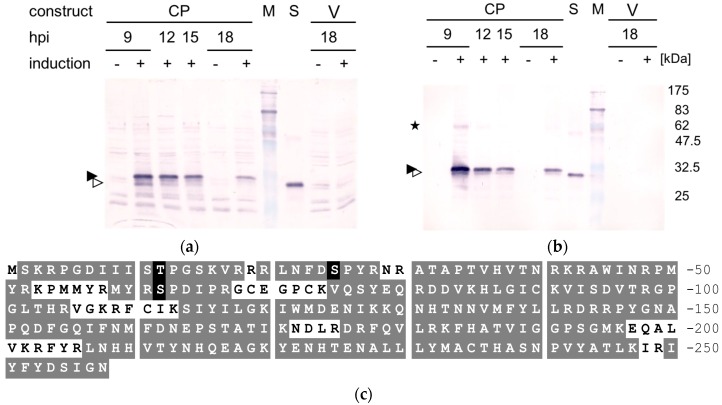

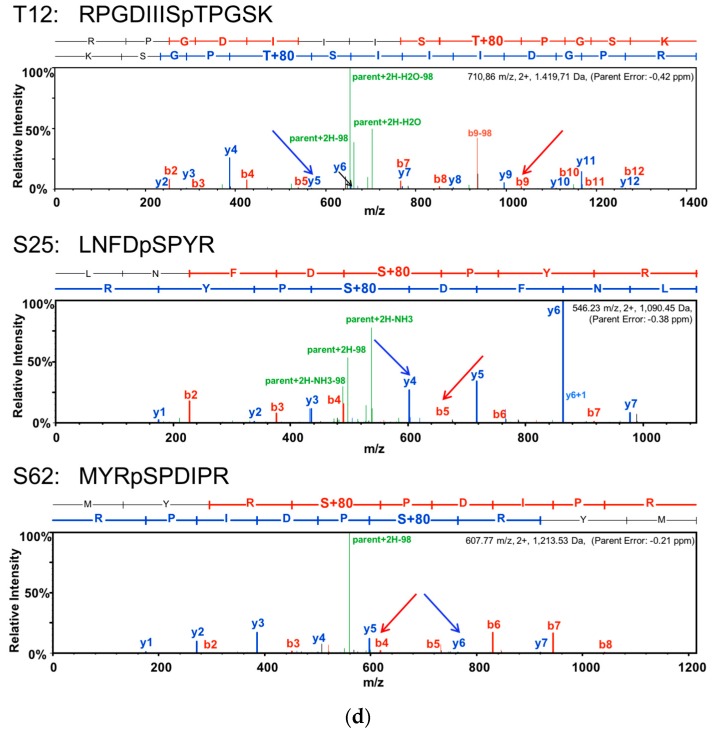
African cassava mosaic virus (ACMV) capsid protein (CP) expression in fission yeast. Cells with pESP1-pREP2:AV1 (CP) or pESP1-pREP2 (V) were harvested at 9, 12, 15 and 18 h post induction (hpi) without thiamine for induction (+) or with thiamine for repression (−). Cell extracts separated into supernatant (**a**) and pellet (**b**) fractions by low-speed centrifugation were analyzed by SDS-polyacrylamide gels (SDS-PAGE) applying 100 µg protein per lane, Western blotted and CP was detected immunologically. Bands of monomeric CP (filled and open arrowheads) and dimers (asterisks) are indicated. Marker proteins (M) with molecular weights (kDa) and standard pCP (S) are compared. (**c**) mass spectrometry results. Amino acid sequence of ACMV CP with amino acids on grey background if unmodified and on black background for modification by phosphorylation. Tryptic peptides with a white background have not been identified; (**d**) mass spectrometric analysis of phosphopeptides. Fragmentation spectra of tryptic peptides containing phosphorylation sites T12, S25, and S62, respectively. Fragments matching the expected sequence are shown in red (b series) and blue (y series) with the peaks corresponding to the fragment ions ending at the phosphorylated residue highlighted by red and blue arrows, respectively.

The ACMV AV1 ORF was transferred into a fission yeast plasmid for expression under the inducible *nmt1* promoter [43]. When induced, yeast expressed CP (yCP) was detected in the supernatant (Figure 1a, arrowheads) as well as in the pellet fractions (Figure 1b, arrowheads) after low-speed centrifugation of cell extracts. Already detectable at 6 hpi in a low amount, the relative proportion of yCP per total protein increased and was maximal between 9 and 12 hpi (Figure 1). Some yCP dimers (Figure 1b, asterisk) resisted treatment with detergents and reducing agents as had been already observed for CP from virus particles previously purified from infected plants (pCP) [17]. yCP was not detected under repressive conditions or in control cells (Figure 1).

A major upper yCP band (Figure 1a,b; filled arrowhead) migrated not as far as pCP (Figure 1a,b; S; open arrowhead). Only considerably fainter bands with higher mobility could be detected for yCP (Figure 1a,b; open arrowhead). The difference in mobility may be caused either by a yeast-specific or a plant-specific modification of the CP. To scrutinize this question, mass spectrometry was employed to examine possible modifications. Tryptic peptides from the gel-purified yCP band from the pellet fraction showed a coverage of 85% for the predicted amino acid sequence inferred from the DNA sequence (AJ427910 of ACMV-NG with a T55M exchange determined by sequencing of the clone used, Figure 1c). A fragment without initiator methionine and serine at the start position indicated that yCP was processed (Figure 1c). Furthermore, T12, S25, and S62 were found to be phosphorylated (Figure 1c,d). These phosphorylation sites were verified by the fragmentation spectra (Figure 1d), whereas fragments with other predicted phosphorylation sites (Y57, Y205) were not resolved.

**Figure 2:**
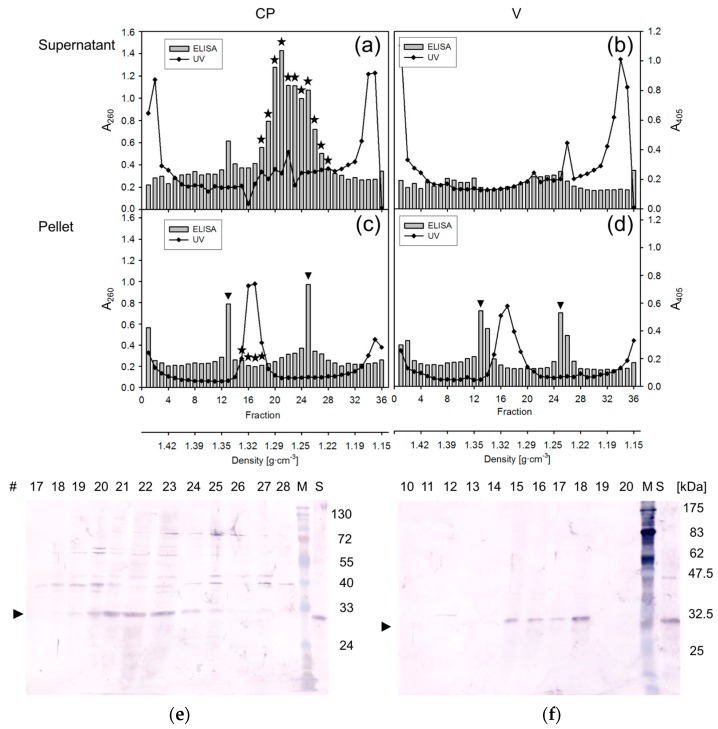
Differentiation of free and DNA-bound CP. Extract supernatants (**a**,**b**) and pellets (**c**,**d**) after isopycnic centrifugation from cells with pESP1-pREP2:AV1 (**a**,**c**, CP) or pESP1-pREP2 (**b**,**d**, V) induced for protein expression and harvested 9 hpi. Profiles for UV absorption (line, A_260_; left *y*-axis) and CP-specific ELISA reads (columns, A_405_; right *y*-axis) are superimposed, and densities are plotted onto a second *x*-axis. Unspecific signals are marked by arrowheads, CP signals confirmed by Western blots (asterisks) on top of the columns; (**e**,**f**) selected fractions analyzed by SDS-PAGE/Western blotting as described in Figure 1.

In order to investigate whether yCP forms complexes with nucleic acids in vivo, supernatants, as well as pellets, from expressing and control cells, were analyzed by isopycnic centrifugation followed by detection of yCP-containing fractions by UV-absorption, ELISA and Western blotting (Figure 2). Supernatant yCP (Figure 2a) migrated to densities (1.21–1.30 g·cm^−3^; fractions #18–28) where the bulk of the yeast proteins was detected by SDS-PAGE and silver staining. The specificity of the ELISA test (Figure 2, columns) was confirmed by Western blotting (Figure 2a, asterisks, Figure 2e), whereas only a low level of unspecific ELISA signals were measured for the control samples (Figure 2b), which yielded no CP-specific bands on Western blots. The pellet samples revealed more unspecific cross-reaction (Figure 2c,d; arrowheads) and low specific signals (Figure 2c; asterisks; #15-18), which were only differentiated by Western blotting (Figure 2f). The corresponding densities (1.30 to 1.33 g·cm^−3^) are typical for virions and minichromosomes [13,17,44,45], as well as host chromatin, as indicated by the UV absorption peak for yCP expressing and control cells (Figure 2c,d; lines).

Several attempts were undertaken to solubilize yCP from the pellet fractions by changing the salt concentrations or pH values. As determined by ELISA, about 50% could be solubilized between 0.4 and 1.0 M NaCl, and less at higher salt concentrations, or about 30% between pH 7.0 and 8.0 (tested range: 4.4–8.1; data not shown). The oligomerization state of the released CP has not been determined, but it is possible that different buffer conditions (salt, salt concentration, pH) may influence the interaction capacity of the CP.

**Figure 3:**
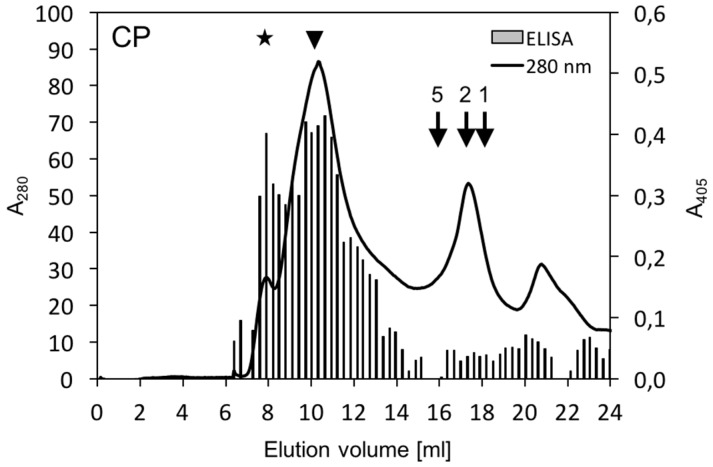
Oligomerization states of CP. The extract supernatants from CP-expressing (CP) or control cells separated by Superose 6 size exclusion chromatography were analyzed as described in Figure 2. ELISA readings of the control were subtracted from CP sample. Arrows indicate expected elution volumes for CP monomers, dimers and pentamers (1, 2, 5) at 18.0, 17.1 and 16.0 mL, respectively. CP signals in the void volume (asterisk) and corresponding to molecular masses of 2.8–6.7 MDa (arrowhead; elution volume of 9.6–11.1 mL) are marked.

In order to determine the oligomerization state, size-exclusion chromatography of supernatant yCP was employed. In initial experiments using Superdex 200 columns, yCP eluted mainly in the void volume. Therefore, columns with larger pore size (Superose 6 HR 10/30) were used further on, with 0.6–0.7 M NaCl in the elution buffer to reduce DNA-binding (Figure 3). Most of the yCP eluted at volumes corresponding to high molecular masses of 2.8–6.7 MDa as well as in the void volume, (Figure 3, arrowhead and asterisk, respectively). Electron microscopic examination of various yCP positive fractions did not reveal any regular structure resembling geminate particles.

**Figure 4:**
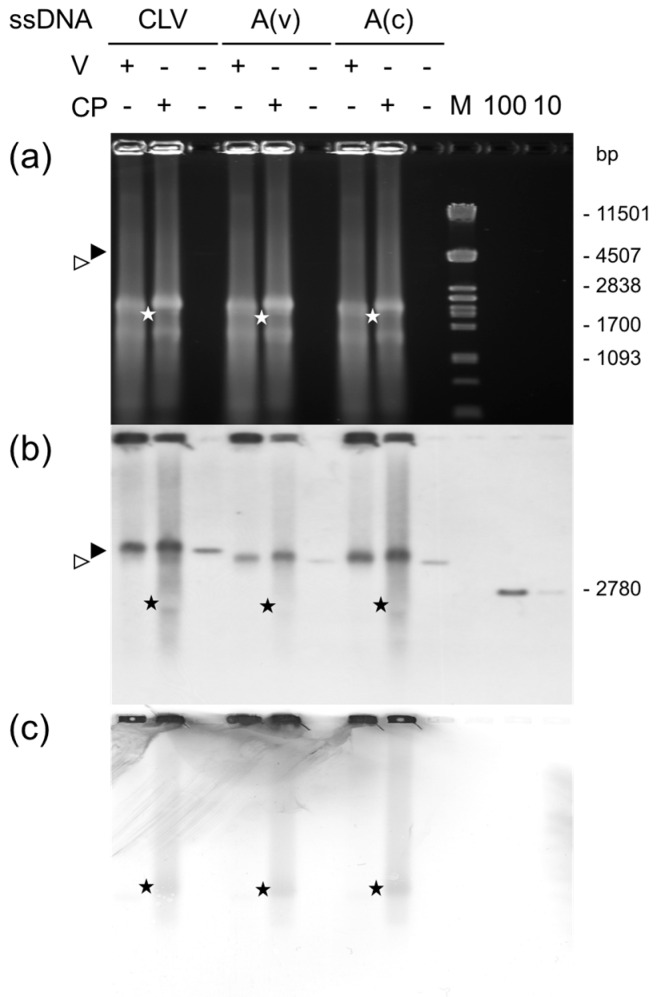
Interaction of CP with ssDNA. Extract supernatants from CP-expressing (CP) or control cells (V) were incubated with ssDNA from pCLV1.3A (CLV), pBlue:ACMVA(v) (A(v)), or pBlue:ACMVA(c) (A(c)). Complexes or ssDNA alone were precipitated with polyethylene glycol and analyzed on an ethidium bromide stained 0.7% agarose gel (**a**) or, after blotting onto nylon (**b**) or nitrocellulose (**c**) membranes, for viral DNA or CP detection using hybridization with an ACMV DNA A-specific probe (**b**) or an CP-specific antibody (**c**), respectively. The corresponding positions of complexes with ssDNA and CP are indicated by asterisks, the positions of ssDNA by arrowheads. M: *Pst*I fragments of λ DNA with bp indicated. Hybridization standards: 100 and 10 pg of linearized full-length ACMV DNA A (10, 100). Bands in (**a**): rRNA.

Next, the capacity of supernatant yCP to interact with DNA was investigated. Binding assays utilizing phage-derived circular ssDNA comprising ACMV DNA A sequences in viral sense [pCLV1.3A and pBlue:ACMVA(v)] or complementary sense [pBlue:ACMVA(c)] orientation were performed. The products were separated on native agarose gels to detect the DNA by hybridization (Figure 4b) and the protein by Western blotting (Figure 4c). The ssDNAs were retarded by yCP-containing extracts, as well as by control extracts (Figure 4b; CP+/V+), but slightly more with yCP. More significantly, however, some DNA was accelerated specifically in the presence of yCP (Figure 4b; smear below the main band). This DNA reached a position (Figure 4b, asterisk) where the hybridization signal appeared negative and the immunological signal showed selectively a band (Figure 4c; asterisk). To find the viral protein at this position is striking, since it discriminates a possible complex formation from an interference with electrophoretic mobility caused primarily by the charges of a basic protein and DNA as discussed previously [21]. To find a yCP band moving together with the DNAs implies the migration of a more compact structure. The negative signal image for the DNA (Figure 4b) is probably caused by the less efficient binding of the DNA to the membrane in the presence of yCP. No preference of yCP for the viral strand compared to the complementary strand was observed in this assay (Figure 4).

**Figure 5:**
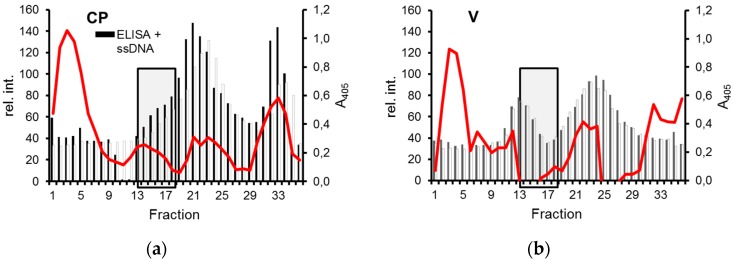
Separation of CP and ssDNA by density gradient centrifugation. Extract pellets either from CP-expressing (**a**) or control cells (**b**) were incubated with ((+)ssDNA) or without ((−)ssDNA) pBlue:CR250 ssDNA, and analyzed in Cs_2_SO_4_ gradients by ELISA for CP as described in Figure 2 and by dot blot hybridization for DNA. Relative hybridization signal intensities (rel. int., red lines) are shown after background subtraction on the left y axis, ELISA values on the right axis. Fractions with relevant differences are boxed.

The sizes of the tested ssDNAs were larger than two viral genomic components, possibly too large to form proper twin-particles with yCP. Therefore, a phagemid was engineered that yields circular ssDNA with genomic size containing the viral CR, which might be important for assembly. In a further assay format, these DNAs were incubated with yeast pellet fractions, and separated by isopycnic centrifugation. Fractions were analyzed by dot blot hybridization and ELISA to detect ssDNA and CP, respectively, in order to determine whether a portion of the ssDNA was shifted to the expected position of gemini-like complexes in the presence of yCP (Figure 5, lines, boxes). Complementarily, a portion of yCP should be shifted to the same position. Indeed, both were the case (Figure 5; #14–18; boxed) if yCP was present. Even more important is the notion that these fractions had the densities (1.29–1.33 g·cm^−3^) where geminiviruses accumulate typically. Extended electron microscopy revealed, however, only pleiomorphic particles, but no twin particles in these fractions (data not shown).

In summary, the results suggest that fission yeast is a suitable host for proper CP expression, which is able to form peculiar complexes with ssDNA, but assembling the intriguing structure of gemini particles obviously needs further processes and/or conditions.

## 4. Discussion

The successful expression of ACMV CP in fission yeast extends the list of several geminiviral proteins that were produced in this organism [35,36,46,47,48,49]. *S. pombe* is a more suitable model organism than *Saccharomyces cerevisiae* and bacteria because its molecular mechanisms are more closely related to those of plants concerning post-translational modifications, chromatin structure, and RNA processing [50,51,52,53,54,55]. In particular, the nuclear environment for CP targeting was thought to be the proper compartment to mimic the situation during plant infection [28]. Since CP was expected to bind sequence-unspecifically to DNA [21,22,23,24], which could interfere with cell division, an inducible promoter allowed a regulated expression to minimize deleterious effects. However, later during cell culture (18–20 hpi), non-expressing cells tend to overgrow the expressing cells leading to lower relative yields of protein. The optimized expression protocol yielded best results at 9 hpi in a broad series of experiments. In contrast to bacterial expression, which leads to inclusion body formation, substantial amounts remained in the supernatant after low-speed centrifugation of cell extracts in fission yeast (Figure 1) and remained in a free state during isopycnic centrifugation (Figure 2). The corresponding lack of the necessity to renaturate the target proteins is likely the most important advantage.

Unexpectedly, yCP formed an electrophoresis band with a lower mobility than pCP. Mass spectrometry confirmed that the authentic protein according to the AV1 ORF was expressed. However, phosphorylation sites were detected for the first time for any geminiviral CP and mapped to at least three positions within the N-terminus (Figure 1). These modifications might be responsible for the mobility shifts in SDS-PAGE. A CP double-band has been described in virus particles purified from *Malva parviflora* for AbMV [56], later identified as a multitude of Sida micrantha mosaic viruses [57,58,59], and for TYLCV from tomato, but not from *N. benthamiana* or *Datura stramonium* plants [60,61]. These results may hint at a differential processing of CP in different plant species. In a survey of ACMV during the course of infection (7–35 dpi) in *N. benthamiana* plants, only one CP-specific band with the smaller apparent molecular weight of 30 kDa (compared to 32 kDa for the upper band) was detectable in total protein extracts [unpublished data, 62], such as the one documented here (Figure 1; S).

Phosphorylation of geminiviral CPs have been described for AbMV after expression in *E. coli* [34]. The cabbage leaf curl virus (CLCuV) CP was acetylated in vitro by an acetyltransferase from *Arabidopsis* initially identified as an interactor of the viral nuclear shuttle protein (NSP) in a yeast two hybrid assay [63]. The authors proposed that the acetylated CP will no longer bind ssDNA, allowing NSP to replace CP on viral DNA for the export of the genome from the nucleus and movement within the plant. However, the mass spectrometry of ACMV yCP did not detect this modification.

It is well-known that geminiviral CPs bind ssDNA as well as dsDNA in a highly cooperative and sequence non-specific manner when expressed in *E. coli* [21,23,24,25] or by in vitro transcription and translation [22]. Nevertheless, no geminivirus-like particles have been reported to assemble in these experiments, and mobility shift assays have to be interpreted with caution due to the basic isoelectric point (pI) of CP [21,24,25]. Expression in fission yeast yielded for the first time sufficiently soluble ACMV CP in supernatant fractions which accumulated in gradient fractions (Figure 3a) with densities (Figure 2) where most host proteins are usually found, but also some virus particles from infected plants [13]. In pellet fractions (Figure 2c; asterisks), CP appeared at higher densities, comparable to those usually found for the main pool of geminivirus particles and chromatin [13,17,44,45]. A smaller amount of yCP may package yeast DNA in complexes that are stable enough for the high Cs_2_SO_4_ salt concentrations in the gradient.

Similarly, the in vitro binding assays (Figure 4 and Figure 5) revealed for the first time particular yCP complexes with ssDNA constructs. The novel observation that yCP accelerates circular ssDNA indicates that it compacts the DNA molecules in addition to a mere binding due to its basic pI. Thus, these complexes might be early steps of assembly, where ssDNA is forced into a certain secondary structure. The bending of DNA induced by binding of a protein can strongly influence the mobility of the nucleoprotein complex, as shown for linear dsDNA fragments [64]. Interestingly, the position of the bend within the DNA affected the electrophoretic migration behavior [64,65]. Complexing of ACMV yCP on ssDNA was observed for the viral and the complementary strand of the ACMV DNA A as well as constructs including only the ACMV CR (Figure 4 and Figure 5), underscoring the largely sequence non-specific DNA binding.

One prerequisite of proper twin particle formation may be the presence of preformed CP pentamers as found after the disassembly described previously [17]. Therefore, size-exclusion chromatography was employed to determine the oligomerization state of yCP. After having varied many conditions to optimize the separation, the final result (Figure 3) revealed higher order assemblies of yCP rather than mono, di- or pentamers, irrespectively of whether DNase treatment or DTT as a reducing agent was used or not. These results may indicate a high potential of yCP for self-interaction, its interaction with yeast proteins or residual yeast nucleic acids protected by yCP against DNase digestion. Of the pellet fractions, about 50% of the yCP was solubilized by the addition of salt. Similarly, SqLCV CP translated in vitro dissociated from ssDNA and dsDNA coupled to cellulose under salt conditions between 0.4 and 1 M KCl [22].

Our results provided one important step further to the assembly of particles from nucleic acids and geminiviral CP. However, extended searches for twin particles in negatively stained samples failed to detect them. Instead, pleiomorphic structures were prevalent in the most promising density gradient fractions. Although the yCP was properly expressed as inferred from mass spectrometry (Figure 1), plant-specific processing or folding may be necessary to build the intriguing structure of geminivirions. Alternatively, this structure may only be formed during rolling circle replication. So far, no empty capsids have been found for geminiviruses and ssDNA may chaperone the assembly process. The binding of MYMIV CP to Rep [20] may be a further hint in this direction. Recently, Wang et al. [66] succeeded in expressing soluble CP in *E. coli* as a fusion with a maltose binding protein, but the purified protein was not tested for DNA binding. For MYMIV, an expression of full-length CP was not successful and only an N-terminal truncated version was obtained in inclusion bodies [20]. Binding studies with cotton leaf curl Kokhran virus-Dabawali (ClCuKV-Dab) CP with extensions both at the N-terminus and the C-terminus have been performed [25]. In case of ACMV, even short deletions within the N-terminus or extensions of either the N-terminus or the C-terminus of the CP impeded particle formation [29].

The difference between yCP and pCP was obvious from the mobility difference. How the conformation or the pCP structure is changed remains to be elucidated, but this might be a critical step to assemble geminivirions properly. The yCP may provide a unique and useful tool in future work to unravel this process in plants.
